# Understanding covariate shift in model performance

**DOI:** 10.12688/f1000research.8317.3

**Published:** 2016-10-17

**Authors:** Georgia McGaughey, W. Patrick Walters, Brian Goldman

**Affiliations:** 1 Modeling & Informatics, Vertex Pharmaceuticals, Boston, MA, USA

**Keywords:** covariate shift, model building, ChEMBL, logistic regression, k-NN

## Abstract

Three (3) different methods (logistic regression, covariate shift and k-NN) were applied to five (5) internal datasets and one (1) external, publically available dataset where covariate shift existed. In all cases, k-NN’s performance was inferior to either logistic regression or covariate shift. Surprisingly, there was no obvious advantage for using covariate shift to reweight the training data in the examined datasets.

## Introduction

A common prerequisite in supervised learning algorithms is that the training and prediction data arise from the same distribution and are independently and identically distributed ( ***iid***) ^1^. Intuitively this is justified, as one should not expect to learn a classifier on one distribution of examples and apply it to accurately predict labels of examples drawn from a different distribution. Covariate shift is a machine learning technique that can be utilized in supervised learning when the training and prediction distributions are known to differ, but the concept being learned remains stationary. While standard machine learning classifiers are trained and then used to predict on arbitrary compounds, covariate shifted classifiers must be trained specifically for each prediction dataset. This is because covariate shifted classifiers weight the training distribution to be more similar to the prediction distribution. A recent book provides an excellent overview of the current state of the art in covariate shift methods ^2^.

Covariate shift frequently occurs during the drug discovery process where learning systems are built to predict physiochemical properties of interest. Initially a chemistry team may focus on a particular chemical series, and information from this series is used to train a learning system. As the project progresses, the chemistry team may refocus their efforts on a new, structurally distinct series. The accuracy of prospective computational predictions on the new series may be compromised as these molecules originate from a distribution that is distinct from the molecular set used to train the learning tool.

For example one may wish to build a learning system to predict hERG activity (unwanted cardiovascular toxicity). Initially the computational tool is trained using series A but must now predict on series B. The concept “binding to hERG” is fixed, however the area of interest has transitioned from chemical series A to chemical series B. The feature vectors describing these two sets are likely related but potentially different; and as such, their covariates have shifted. Put more mathematically, the probability of observing a feature vector from the prediction set is different from the probability of observing a feature vector from the training set. That is, the training and prediction sets are ***non-iid***. A well-constructed learning system will recognize that predictions on series B are outside the “domain of applicability” of the model and predict with low confidence. The covariate-shift method attempts to adjust the domain of applicability so that it is more aligned with the prediction set. It is analogous to a nearest neighbor classifier but employs distributions rather than individual examples. Covariate shifted classifiers weight examples from the training set to create a distribution that is more aligned with the prediction set. This weighted data set is then used to train the classifier, resulting in a covariate shifted classifier. As such, covariate shift is applied at the distribution level whereas nearest neighbor methods are applied at the example level. Once a training set has been shifted, it can be used by any machine learning algorithm.

Covariate shift methods typically reweight instances in the training data so that the distribution of training instances is more closely aligned with the distribution of instances in the prediction set. This is accomplished by providing more weighting during model building to an instance in the training set that are similar to an instance in the prediction set. It has been shown ^3^ that the appropriate importance weighting factor w(x) for each instance “x” in the training set is:

$$w(x)=\frac{p_{p}(x)}{p_{t}(x)}\;(1)$$

where *p _t_* ( *x*) is the probability of seeing instance x in the training set and *p _p_* ( *x*) is the probability of seeing x in the prediction set. It is important to note that only the feature vector values (not their labels) are used in reweighting. The importance weighting scheme is intuitively understandable. If the probability of seeing a particular instance from the training set in the prediction is very small, then this instance should carry little weight during the training process and consequently have little effect on the decision function.

**Figure 1.**
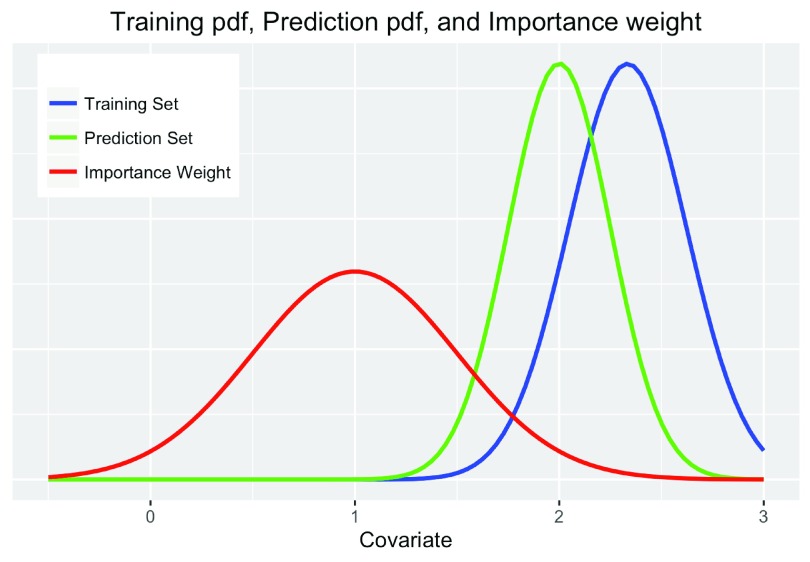
Train, prediction and importance.

Figure 1 plots two Gaussian distributions and w(x). If instances from the blue distribution are used for training a classifier to predict on an instance from the green distribution then the red curve gives the importance of each instance. Note the increased importance for instances from the training distribution overlapping with high-density regions of the prediction distribution.

## Methods

For our experiments, we use a logistic regression classifier where each training instance is weighed by its importance w(x). For the calculation of w(x) we use the Kullback-Leibler Importance Estimation Procedure (KLIEP) method developed by Sugiyama ^4^. The KLIEP method is based on the Kullback-Leibler divergence theorem and attempts to find weights to minimize the divergence from p _*train*_(x) to p _*predic*t_(x). Briefly, the importance is modeled as a linear function:

$$\overset{⌢}{w}(x)=\sum_{i=1}^{b}\alpha_{i}*\varphi_{i}(x)\;(2)$$

The *α _i_* are the weights to be learned and *φ _i_* the basis functions. The importance weight from Equation 1 can be rearranged and used to estimate the probability of observing a feature vector in the predictive set.

$${\hat{p}}_{p}(x)=w(x)p_{t}(x)\;(3)$$

The KL divergence from *p _p_*( *x*) to its estimate ${\hat{p}}_{p}(x)$ can then be expressed as:

$$KL[p_{p}(x)||{\hat{p}}_{p}(x)]=\int p_{p}(x)\;\log\left(\frac{p_{p}(x)}{p_{t}(x)\overset{⌢}{w}(x)}\right)dx$$

After algebraic manipulation, removing terms independent of $\hat{w}(x)$ and adding constraints to ensure proper normalization, a final objective function to be maximized can be derived as (see 4 for details):

$$\begin{array}{c} \begin{array}{cc} \begin{array}{l} \max imize\\ {\left\{\alpha_{l}\right\}}_{l=1}^{b} \end{array} & \left[\sum_{j=1}^{n_{p}}\log\left(\sum_{l=1}^{b}\alpha_{l}\varphi_{l}(x_{j})\right)\right] \end{array}\\ \begin{array}{l} subject\;to:\sum_{j=1}^{n_{t}}\sum_{l=1}^{b}\alpha_{l}\varphi_{l}(x_{j})=1\\ and\;\alpha_{1},\alpha_{2},\ldots ,\alpha_{b}\geq 0 \end{array} \end{array}$$

The resulting problem is convex and can be solved using standard optimization techniques. The result is an expression for w(x) that allows calculating weights for a training instance x. These weights can then be incorporated when training a classifier to obtain a covariate shifted version of the classifier.

## Toy example

**Figure 2.**
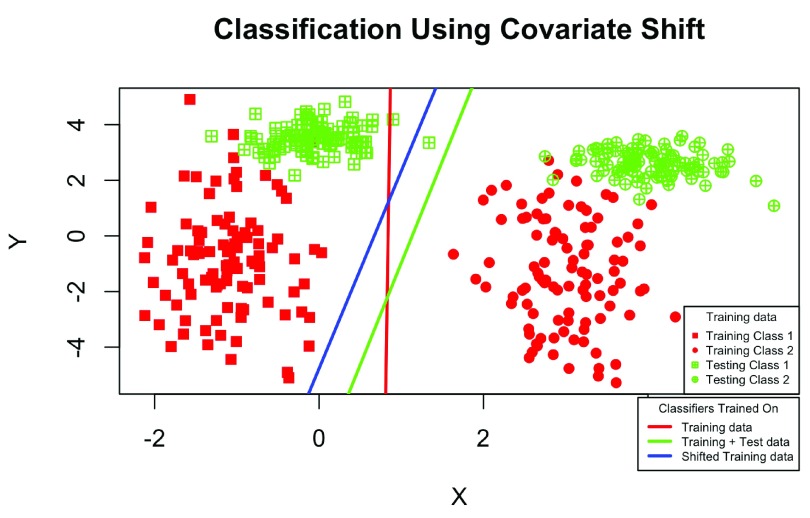
Classification using covariate shift.

To demonstrate the use of covariate shift methods, we repeated a simple toy experiment as detailed in 3. Figure 2 graphically displays the results we obtained.

The red training points are drawn from two (2) two-dimensional Gaussian distributions representing a class 1 and a class 2. The green prediction points are drawn from a slightly rotated version of the training distributions. The red line plots the classifier obtained when training on only the training points; the green line plots the classifier trained on both the training and prediction points (the optimal classifier in this case). The blue line plots the classifier trained on the training data that was weighted by the importance factor as estimated by the KLIEP method. Note how the blue line is shifted towards the optimal classifier, demonstrating the effect of the KLIEP algorithm and covariate shift.

## Experiments

The beta secretase IC _50_ data derived from the ChEMBL database Units are in nM. Click here for additional data file. Copyright: © 2016 McGaughey G et al. 2016 Data associated with the article are available under the terms of the Creative Commons Zero "No rights reserved" data waiver (CC0 1.0 Public domain dedication).

Using the Python programming language we implemented the KLIEP method for determining weights for use in covariate shift ^5^. In principle, covariate shift is applicable to any classifier that allows weighting of input instances (e.g. support vector machines and random forest). For this study we wanted to isolate the effects of covariate shift and therefore selected a classifier without adjustable parameters and used logistic regression (LR). Logistic regression is a classification technique analogous to linear regression and is applicable when the dependent variable is categorical ^6^. We combined logistic regression with KLIEP and applied it to five different in-house ADME (absorption, distribution, metabolism and excretion) assays and one external dataset (beta secretase). The cutoff values for determining the binary categories for the compounds in each dataset are listed in Table 1. Due to inherent noise in the assays we discard data where the assay values are between the positive and negative cutoffs listed in the Table 1. We compare KLIEP+Logistic Regression (KL+LR) to Logistic Regression and a k-NN (using Tanimoto similarity) classifier (k=5).

**Table 1.** Proprietary Assays Utilized for Covariate Shift Analysis.

Data Set	Positive Cutoff	Negative Cutoff
hERG	IC50 <10uM	IC50 > 15uM
Human Liver Microsome (HLM)	stable > 60% remain	unstable < 30% remain
Rat Liver Microsome (RLM)	stable > 60% remain	unstable < 30% remain
Solubility (water)	insoluble < 10uM	soluble > 200uM
Solubility (DMSO)	insoluble < 10uM	soluble > 50uM

Legend: The cutoff values for determining the binary categories (actives or inactives) for the compounds in each dataset are listed.

For each dataset the molecules were sorted by compound registration date. The first 75% of the data comprised the master training set while the remainder formed the master prediction set. Temporal ordering of the data represents the evolving coverage of chemical space by drug discovery projects and consequently captures the natural “shifting” of the covariates. Classifier performance statistics are generated by performing twenty different runs, each on a random 80% of the master files. Performance statistics for each classification task are then obtained by averaging the results of the twenty individual folds. In all cases, OpenEye ^7^ path fingerprints are used as feature vectors. We experimented with different fingerprints provided by OpenEye (MACCS 166 bit structural keys and circular fingerprints) and found that they had no significant effect on the outcome.

**Figure 3.**
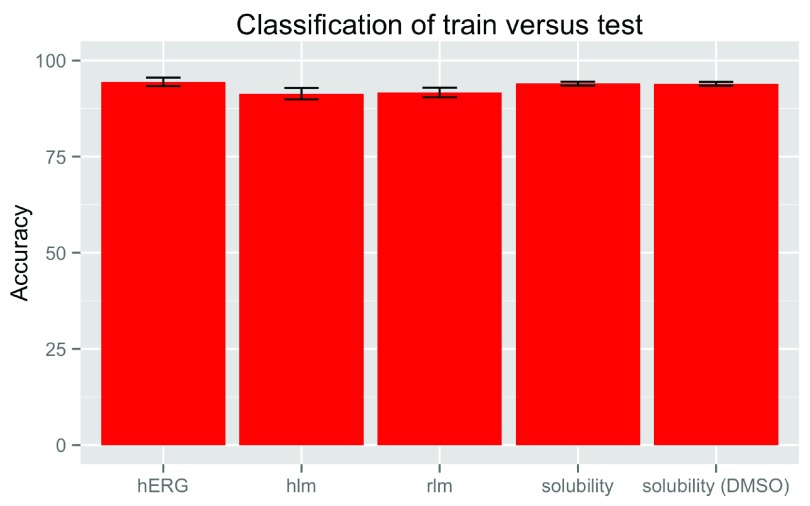
Classification of train versus test.

To ensure the data was amenable to covariate shift we generated classifiers separating “training” from “prediction” data. Figure 3 shows performance of LR on this separation task. For each dataset we are able to compute highly accurate classifiers. This indicates that the training and prediction data are drawn from different distributions and hence are appropriate for covariate shift methods. This is a necessary condition for covariate shift but does not imply model improvement over unweighted data.

**Figure 4.**
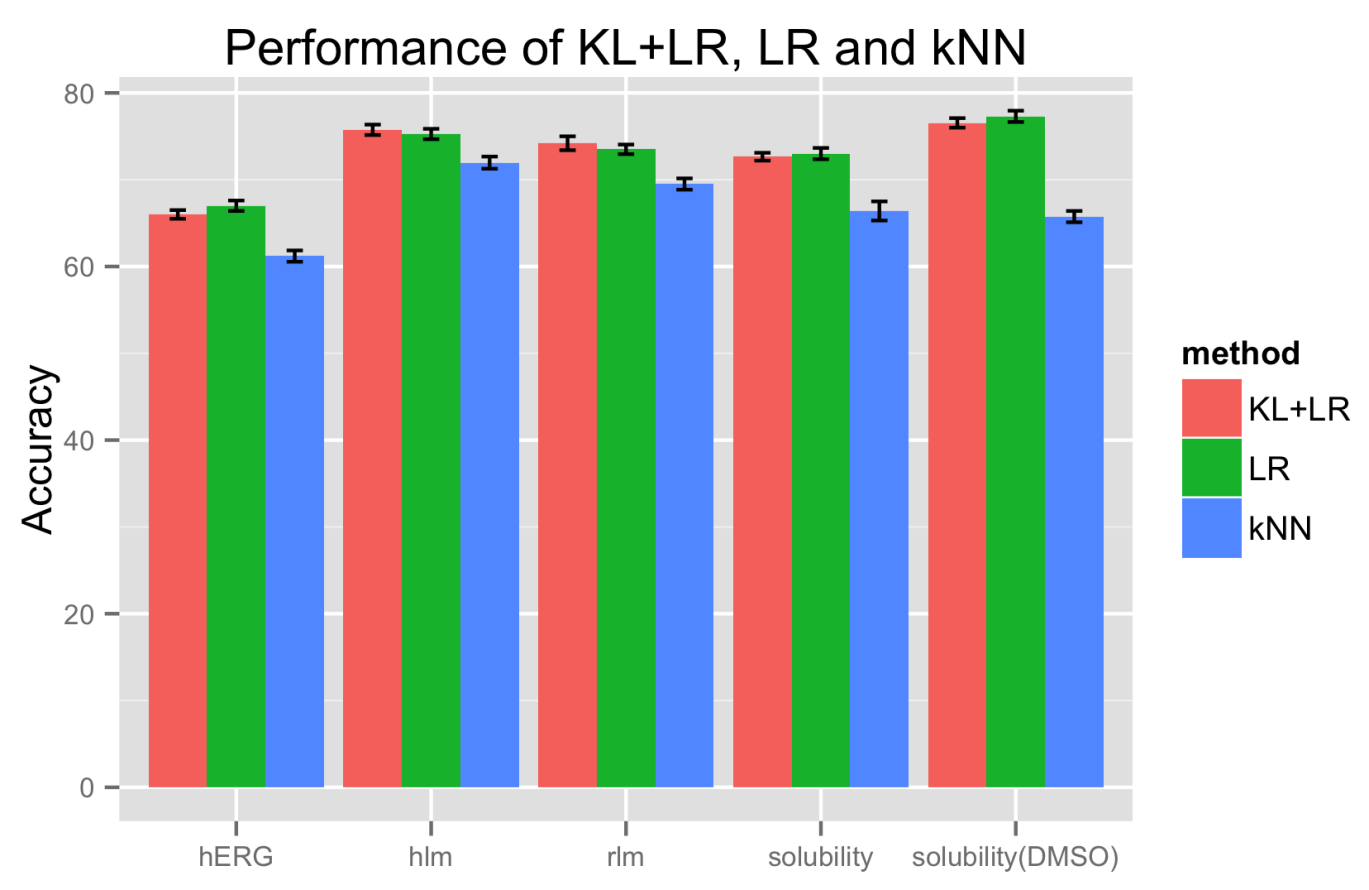
Performance of KL+LR, LR and k-NN.

Figure 4 compares the performance of KL+LR, LR and k-NN on the five (5) datasets. One can see from the graph that KL+LR failed to provide any statistical improvement over standard LR.

We extended the study to include an external dataset provided by ChEMBL ^8, 9^ such that others could use their own fingerprints and independently support or refute our claims. We chose the beta secretase IC _50_ data as it is a well established biochemical screen, highly accurate and contains > 7000 data points crossing multiple orders of magnitude, which are publically available. Using OpenEye path fingerprints and K-Means clustering we clustered the dataset into two clusters, A and B. Under cross-validation, a logistic regression classifier was able to separate the two clusters with a high level of accuracy (90%) indicating that the clustered dataset would be appropriate for application of the covariate shift algorithm. Ten random subsets of molecules from cluster A were used to train a logistic regression classifier using covariate shift which was then used to predict on molecules from cluster B. The performance of the shifted classifier was compared to an unshifted classifier trained and tested on the same clustered datasets and random splits. The process was repeated by training on molecules from cluster B and predicting on molecules from cluster A. Analogous to the internal datasets, as measured by overall classifier accuracy, there was no statistical advantage for application of covariate shift (Shifted Accuracy: 82.95% +/- 1.6%; Unshifted Accuracy 82.73% +/- 1.2%).

**Figure 5.**
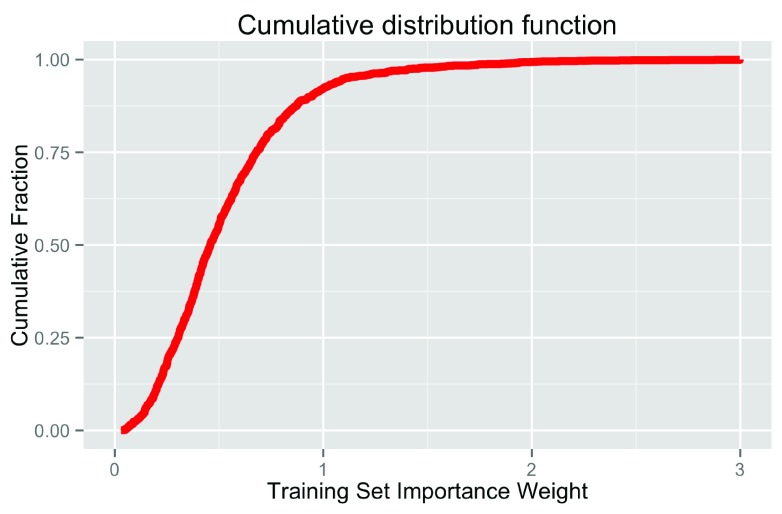
Cumulative distribution function.

A possible explanation for the failure of the covariate shift method to provide a boost in predictive performance could be that the calculated importance weights are all similar. This would cause each training example to exert the same influence on the decision function and thus the importance weighting would have no effect. This was not the case. Figure 5 plots the cumulative distribution function of the importance weight for the training set compound. The plot demonstrates that weights are distributed across a range of classifier performance.

## Conclusions

We have applied the KLIEP method to five (5) internal data sets and one (1) external data set where covariate shift was evident. Although KL+LR was an advantage over k-NN, there is no statistical advantage of reweighting the training dataset. We are surprised with this outcome and are currently exploring other datasets where application of covariate shift may improve the predictions.

## Data availability

The data referenced by this article are under copyright with the following copyright statement: Copyright: © 2016 McGaughey G et al.

Data associated with the article are available under the terms of the Creative Commons Zero "No rights reserved" data waiver (CC0 1.0 Public domain dedication). 

*F1000Research*: Dataset 1. The beta secretase IC _50_ data derived from the ChEMBL database, 10.5256/f1000research.8317.d117882 ^10^
