# Supplementary material for: Understanding covariate shift in model performance
Source: F1000Res. 2016 Oct 17;5:Chem Inf Sci-597. Originally published 2016 Apr 7. [Version 3] doi: 10.12688/f1000research.8317.3 (PMC5070592; doi:10.12688/f1000research.8317.3)
Supplement: Peer review history

# Peer review: Referee response for version 3

**DOI:** 10.5256/f1000research.10228.r17055

**Published:** 2016-10-18

**Authors:** Robert Sheridan

**Affiliations:** 1 Cheminformatics Department, Merck Research Laboratories, Rahway, NJ, USA

The only issue I am still having trouble with is equation 1.

 w(x)=Pp(x)/Pt(x)

 where the P’s represent probabilities for molecule x. Given that the training and test sets are distinct points (molecules) in a chemical descriptor space and the training and test sets do not overlap, how are the probabilities for molecule x calculated from the other molecules?

I have read this submission. I believe that I have an appropriate level of expertise to confirm that it is of an acceptable scientific standard.

# Peer review: Referee response for version 2

**DOI:** 10.5256/f1000research.9428.r14444

**Published:** 2016-06-21

**Authors:** Martin Vogt

**Affiliations:** 1 Department of Life Science Informatics, Bonn-Aachen International Center for Information Technology (B-IT), LIMES (Life & Medical Sciences Institute) Program Unit Chemical Biology and Medicinal Chemistry, University of Bonn, Bonn, Germany

The revised paper has been significantly improved and addresses a number of issues raised in the original reviews. However, a few issues remain that should be addressed.

 1. The description of Figure 1 is inconsistent with the figure legend. It seems that the red and blue labels in the legend of Figure 1 need to be swapped to make the figure consistent with the description in the text: The red and green curves look like pdfs whose integrals are very similar and close to 1 while the blue curve has a much larger area inconsistent with a pdf.

 2. For the ChEMBL data set the inactivity/activity cutoffs used should be mentioned.

I have read this submission. I believe that I have an appropriate level of expertise to confirm that it is of an acceptable scientific standard.

# Peer review: Referee response for version 2

**DOI:** 10.5256/f1000research.9428.r14472

**Published:** 2016-06-20

**Authors:** Robert Sheridan

**Affiliations:** 1 Cheminformatics Department, Merck Research Laboratories, Rahway, NJ, USA

Having read the revised paper it seems better, but I’m still somewhat puzzled about a few things.

  

1. I am getting the feeling that the K-NN method is meant as a baseline control method since by definition K-NN looks at only the training set compounds close to the test set compounds, so there is an implicit selection of training set compounds,  and this should have a similar effect as covariant shift.  This is not explicitly said in the paper.
2. The authors do not  try sophisticated but more “standard” classification methods like random forest or SVM, and don’t say why not.
3. Both myself and the other reviewer seem confused by Figure 1.  The red line is supposed to be the importance weight.  However, it implies that the highest weights are given in a region of descriptor space far away from both training and test sets.

I have read this submission. I believe that I have an appropriate level of expertise to confirm that it is of an acceptable scientific standard, however I have significant reservations, as outlined above.

# Response

**Published:** 2016-06-21

**Authors:** Georgia McGaughey

**Affiliations:** Vertex Pharmaceuticals, USA

I'm responding directly to the questions you posed, in that order.

 1.  Agreed that we could have added such a sentence to the paper.

 2.  We didn't examine more standard classification methods as we were specifically studying whether there was a benefit in using covariate shift. We were not interested in exploring RF or SVM as that was out of scope for this paper.

 3. The three curves in Figure 1 were all drawn as per a Gaussian distribution and we thought it would look odd to have the red line go straight to zero when, for example, x=1.  The purpose of the importance weight being close to 0 (on the y axis) when x=2, for instance, is because there's much overlap between the training and the prediction set.  When, for instance, there isn't much overlap (ie, x=1.5), the importance weight goes up.  I can see why one might be confused when x=1;  that was merely to show that when there's minimal overlap between prediction and training, that the importance weight is quite high.

# Response

**Published:** 2016-08-02

**Authors:** Martin Vogt

**Affiliations:** University of Bonn, Germany

Concerning Figure 1:

 According to equation (1) w(x) = p_p(x)/p_t(x) there is a certain symmetry between the importance weight w(x) and the training pdf p_t(x).

 This means that in Figure 1 the red curve can either show the importance weight and the blue curve the training pdf or vice versa without compromising the accuracy of the figure.  What speaks against interpreting the blue curve as a pdf is that it clearly has a much larger area than the green curve representing the prediction pdf, which should be 1 in both cases. From visual inspection the red curve has an area much closer to 1 than the blue curve and it should thus be interpreted as training pdf while the blue curve represents the importance weight. Even though the figure is only used for illustration purposes it could be improved by either relabelling or by rescaling the red and blue curves accordingly.

# Response

**Published:** 2016-08-03

**Authors:** Georgia McGaughey

**Affiliations:** Vertex Pharmaceuticals, USA

The figure is really meant to be an illustration of the importance weight - and not mathematically accurate with respect to pdfs. In actuality, the green and blue curve look like pdfs but they really are not. The green and blue curves are as such not normalized using the same scales - so the area under each curve does not sum to 1. We could make the blue curve a shifted version of the green curve then recompute the importance weight. What we were hoping to illustrate is that if an example from the training (blue) was pulled at x = 1 it would be very important for training because it is rare and the testing (green) set has non-zero support at x = 1.

 Additionally, another perhaps confusing aspect about the figure is that the y-axis represents two values on different scales: 1) the importance weight and 2) the probability (relative) of seeing a training/test example. We will redraw the figure and upload a new version.

# Peer review: Referee response for version 1

**DOI:** 10.5256/f1000research.8943.r13393

**Published:** 2016-04-25

**Authors:** Martin Vogt

**Affiliations:** 1 Department of Life Science Informatics, Bonn-Aachen International Center for Information Technology (B-IT), LIMES (Life & Medical Sciences Institute) Program Unit Chemical Biology and Medicinal Chemistry, University of Bonn, Bonn, Germany

The study investigates the influence of accounting for covariate shift in classification performance using logistic regression models. Overall, this short paper is very well and clearly written, however the method section should be expanded (see below). Although no increase in performance could be established by accounting for covariate shift, it provides an excellent basis for further investigations.

Suggestions/Corrections:

The method section should be expanded:

1. I assume all models were trained as binary classifiers. This is potentially confusing as the chosen ADME properties in the experimental data could also have been modelled using regression models. This should be stated clearly and explained how labels (good/bad) are assigned to the training instances for the different ADME properties (and how labels are assigned to the ChEMBL data given the potencies).
2. Which basis functions (kernels?) were used in equation (2)?
3. What distance measure was used for k-NN (e.g., Soergel/Tanimoto, Hamming)?
4. In Figure 3 (and 4), given the imbalance in data size between training and test set, consider reporting the balanced accuracy. E.g. a trivial classifier classifying each compound as "training" compound would have an accuracy of 75% based on the imbalance of the data set, which needs to be taken account when interpreting Figure 3.
5. The authors provide a data set for download although they do not explicitly report the results for that data set. The results should be reported.

Typos:

- In the formula for KL on page 3 the two vertical bars should have the same size.
- In Figure 1, the labels for the red and blue line are mixed up.

I have read this submission. I believe that I have an appropriate level of expertise to confirm that it is of an acceptable scientific standard, however I have significant reservations, as outlined above.

# Peer review: Referee response for version 1

**DOI:** 10.5256/f1000research.8943.r13266

**Published:** 2016-04-08

**Authors:** Robert Sheridan

**Affiliations:** 1 Cheminformatics Department, Merck Research Laboratories, Rahway, NJ, USA

This is potentially an important negative result in QSAR, however I think some revision is necessary because some aspects are unclear.

The title “Understanding covariate shift…” is a little weak. One could say “Failure of covariant shift to improve model performance…”

It needs to be explicitly pointed out in the introduction that in most QSAR one builds a model then is able to predict arbitrary compounds. On the other hand, to use covariant shift, one must know which molecules one is predicting before one can generate the model. One can regard “lazy learning” as an extreme version of covariant shift:  neighbors of the test set molecules are given weights of 1.0 and all other molecules are given weights of 0.

I need a little more explanation in words of how the weighting is done for training set compounds. Since we are using substructure descriptors here, I am finding it hard to visualize. For example, are we just using distance to the nearest test set example, or are we looking at overlap of the training set descriptors with the distribution of test set descriptors?

Practically no explanation is given as to what QSAR methods are being used. I know what K-NN is and I presume LR is linear regression. Why weren’t popular methods like random forest, SVM, or PLS tried? 

The color key in Figure 1 does not seem to match what is in the text. In any case, perhaps a better way of looking at would be the enclosed figure.

I have read this submission. I believe that I have an appropriate level of expertise to confirm that it is of an acceptable scientific standard, however I have significant reservations, as outlined above.
